# Imitation, Sign Language Skill and the Developmental Ease of Language Understanding (D-ELU) Model

**DOI:** 10.3389/fpsyg.2016.00107

**Published:** 2016-02-16

**Authors:** Emil Holmer, Mikael Heimann, Mary Rudner

**Affiliations:** ^1^Linnaeus Centre HEAD, Swedish Institute for Disability Research, Department of Behavioural Sciences and Learning, Linköping UniversityLinköping, Sweden; ^2^Swedish Institute for Disability Research and Division of Psychology, Department of Behavioural Sciences and Learning, Linköping UniversityLinköping, Sweden

**Keywords:** imitation, sign language, manual gesture, representation, development

## Abstract

Imitation and language processing are closely connected. According to the Ease of Language Understanding (ELU) model (Rönnberg et al., 2013) pre-existing mental representation of lexical items facilitates language understanding. Thus, imitation of manual gestures is likely to be enhanced by experience of sign language. We tested this by eliciting imitation of manual gestures from deaf and hard-of-hearing (DHH) signing and hearing non-signing children at a similar level of language and cognitive development. We predicted that the DHH signing children would be better at imitating gestures lexicalized in their own sign language (Swedish Sign Language, SSL) than unfamiliar British Sign Language (BSL) signs, and that both groups would be better at imitating lexical signs (SSL and BSL) than non-signs. We also predicted that the hearing non-signing children would perform worse than DHH signing children with all types of gestures the first time (T1) we elicited imitation, but that the performance gap between groups would be reduced when imitation was elicited a second time (T2). Finally, we predicted that imitation performance on both occasions would be associated with linguistic skills, especially in the manual modality. A split-plot repeated measures ANOVA demonstrated that DHH signers imitated manual gestures with greater precision than non-signing children when imitation was elicited the second but not the first time. Manual gestures were easier to imitate for both groups when they were lexicalized than when they were not; but there was no difference in performance between familiar and unfamiliar gestures. For both groups, language skills at T1 predicted imitation at T2. Specifically, for DHH children, word reading skills, comprehension and phonological awareness of sign language predicted imitation at T2. For the hearing participants, language comprehension predicted imitation at T2, even after the effects of working memory capacity and motor skills were taken into account. These results demonstrate that experience of sign language enhances the ability to imitate manual gestures once representations have been established, and suggest that the inherent motor patterns of lexical manual gestures are better suited for representation than those of non-signs. This set of findings prompts a developmental version of the ELU model, D-ELU.

## Introduction

There is a close connection between mental representation and imitation, the behavioral repetition of another person’s act (Brass and Heyes, 2005). In particular, there are empirical indications of a relationship between imitation of manual gestures and both lexical representation (McEwen et al., 2007) and language comprehension (Farrant et al., 2011). For sign language users, manual gestures may bear phonological and semantic information. Indeed, it has been shown that the ability to imitate manual gestures is related to gesture-based phonological representation in deaf signing children (Mann et al., 2010). However, it is not known whether the ability to imitate manual gestures is related to existing semantic representations in this group. In the present study, we investigated whether knowledge of Swedish Sign Language (SSL) is related to the ability to imitate manual gestures that are familiar (lexical items in SSL), unfamiliar (lexical items in British Sign Language, BSL), or illegal (non-signs), in children whose language skills are still developing.

Sign languages are natural languages that are performed in the manual–visual modality and include sublexical, lexical, and syntactic structures analogous to spoken languages (for a review, see Emmorey, 2002). Whereas, the sublexical structure of spoken languages is based on the patterning of speech sounds, the sublexical structure of sign languages is based on the patterning of a number of articulatory parameters including: formation and orientation of the hands; finger or/and hand movements; placement of the hand(s) in relation to the body; and non-manual facial gestures (Brentari, 2011). Thus, for deaf and hard-of hearing (DHH) signing children, manual gestures are sometimes linguistic and may bear semantic and phonological information. Even when a manual gesture is not part of the lexicon, its formational characteristics may be similar to those of lexicalized signs, or even qualify it as a potentially lexicalized sign. However, for hearing non-signing children, manual gestures only involve motoric information, unless they are emblematic, e.g., “thumbs up”. In the present study, participants imitated signs that were lexicalized in SSL or BSL, and non-emblematic non-signs. For Swedish DHH signing participants the SSL signs bore both semantic and phonological information, while BSL signs bore phonological information only. For hearing non-signing participants, neither SSL nor BSL signs bore either semantic or phonological information. Non-signs bore no semantic information for either group and only reduced phonological information for the signing group.

The Ease of Language Understanding (ELU) model (Rönnberg, 2003; Rönnberg et al., 2008, 2013) describes how language understanding depends on pre-existing representations. The model states that language processing is rapid and automatic if input matches pre-existing phonological and semantic representations (Rönnberg et al., 2013) and it is likely that the best match is obtained when phonological and semantic representations are available simultaneously. When only matching phonological representations are available, a cohort of lexical candidates will be activated (Marslen-Wilson, 1987) that is unconstrained by meaning, and language processing will probably be less efficient. When input bears reduced phonological information, phonological constraints will be fewer and processing will probably be even less efficient (Rudner et al., 2016). These factors are likely to be of importance even in the developing language system (Mann et al., 2010; Sundström et al., 2014). Thus, in the present study, we predicted that Swedish DHH signing children would be better at imitating SSL signs with both semantic and phonological information than BSL signs with phonological information only, and better at imitating lexical signs (SSL and BSL) than non-signs with reduced phonological information. Because recent studies indicate that non-signs are more difficult to process than lexical signs, even for non-signers (Cardin et al., 2016; Rudner et al., 2016), we predicted that both groups would be better at imitating lexicalized signs (both SSL and BSL) than non-signs.

We also predicted that initially the hearing non-signing children would be worse at imitating all types of manual gestures than DHH signing children at a similar developmental level. This prediction was based on the former group’s limited experience of signs with linguistic and symbolic information. However, we predicted that the act of imitation would help establish representations (Brass and Heyes, 2005) of the manual gestures and, thus, that the performance gap between groups would narrow when imitation was elicited a second time. Moreover, we predicted that imitation performance on both occasions would be associated with linguistic skills (McEwen et al., 2007), especially in the manual modality (Mann et al., 2010).

## Materials and Methods

### Participants

#### Deaf and Hard-of-Hearing Participants

All five of the Swedish state special schools for deaf and hard-of-hearing (DHH) pupils were invited to be part of this study. In these schools, pupils are taught in both SSL and spoken and/or written Swedish and admission is granted for children with hearing impairment. Two schools agreed to participate. Staff members identified seventeen potential participants who showed an interest in text and were able to read words at a level corresponding to typical readers in Grade 1. Pupils attending Swedish state primary schools for DHH children represent a heterogeneous population (Svartholm, 2010), which was also reflected in the sample. Four potential participants had an additional severe medical or developmental disability and were thus excluded: 13 DHH pupils (seven girls) with a mean age of 10.2 years (*SD* = 2.3) and attending grades 1–7 at the first testing occasion were included in the present study. Eleven used technical aids: five used only hearing aid (HA) (four bilateral); five used only cochlear implant (CI) (four bilateral), and one had a CI on one ear and a HA on the other. Up-to-date audiological records were not available and since imitative ability, and its relationship with language and cognitive skills, was the focus of this study, audiological measurements were not made. Two participants had a vision deficit which was corrected. All participants used SSL: nine as their primary language (mean age of first exposure to SSL = 2.8 years, *SD* = 3.3, range 0.0–8.0; *n* = 6), four of whom had at least one deaf native signing parent; the other four used SSL in school and occasionally at home and during spare time activities (mean age of first exposure to SSL = 6.1 years, *SD* = 4.0, range 3.0–11.7). Seven participants were born abroad; age at which residence in Sweden commenced ranged from 2.2 to 10.6 years (*n* = 5). Non-verbal intelligence (NVIQ) of participants was screened using Raven’s Colored Progressive Matrices (CPM) (Raven and Raven, 1994); twelve participants scored between the 5th and 95th percentile, and one was one point below (*M* = 25.2, *SD* = 5.88). Three families omitted to provide background data in full or in part.

#### Hearing Participants

Thirty-six typically developing children (20 girls) with no reported hearing impairment or knowledge of sign language attending first grade of primary school took part. In grade one, typically developing children are starting to learn to read. They were sampled from four different schools in a municipality in southeast Sweden with representative socioeconomic status. The mean age of the participants at the first occasion was 7.5 years (*SD* = 0.3). Swedish was their first language. One had corrected to normal vision. NVIQ of the participants was screened using Raven’s CPM (Raven and Raven, 1994) and all scored between the 5th and 95th percentile (*M* = 25.4; *SD* = 4.35).

### Procedure

All participants were tested individually at their school on two occasions (T1 and T2) separated by 35 weeks. Hearing participants were instructed in Swedish, and DHH children were instructed in their preferred communication mode. Instructions in SSL were provided by a test leader fluent in SSL, and were based on a rephrased version of the Swedish instructions in SSL following a formal coding system (Bergman, 2012). The SSL instructions were coded by a deaf native SSL user, and checked by three of the test leaders in the study. For practical reasons, test order was individually adapted and breaks were taken when needed; however, hearing participants did the imitation task as the second task and DHH participants did it as one of the last four tasks on both occasions. This study is part of a larger project, and data relating to predictor variables in the present study were collected at T1 and reported in Holmer et al. (2016). Test leaders made sure that the participant understood each task before testing took place, and participants practiced all tasks except the imitation task before administration. The present study was approved by the regional ethical review board and all participants and their parents gave informed consent which was attested in writing by the parents.

### Imitation of Manual Gestures

**FIGURE 1:**
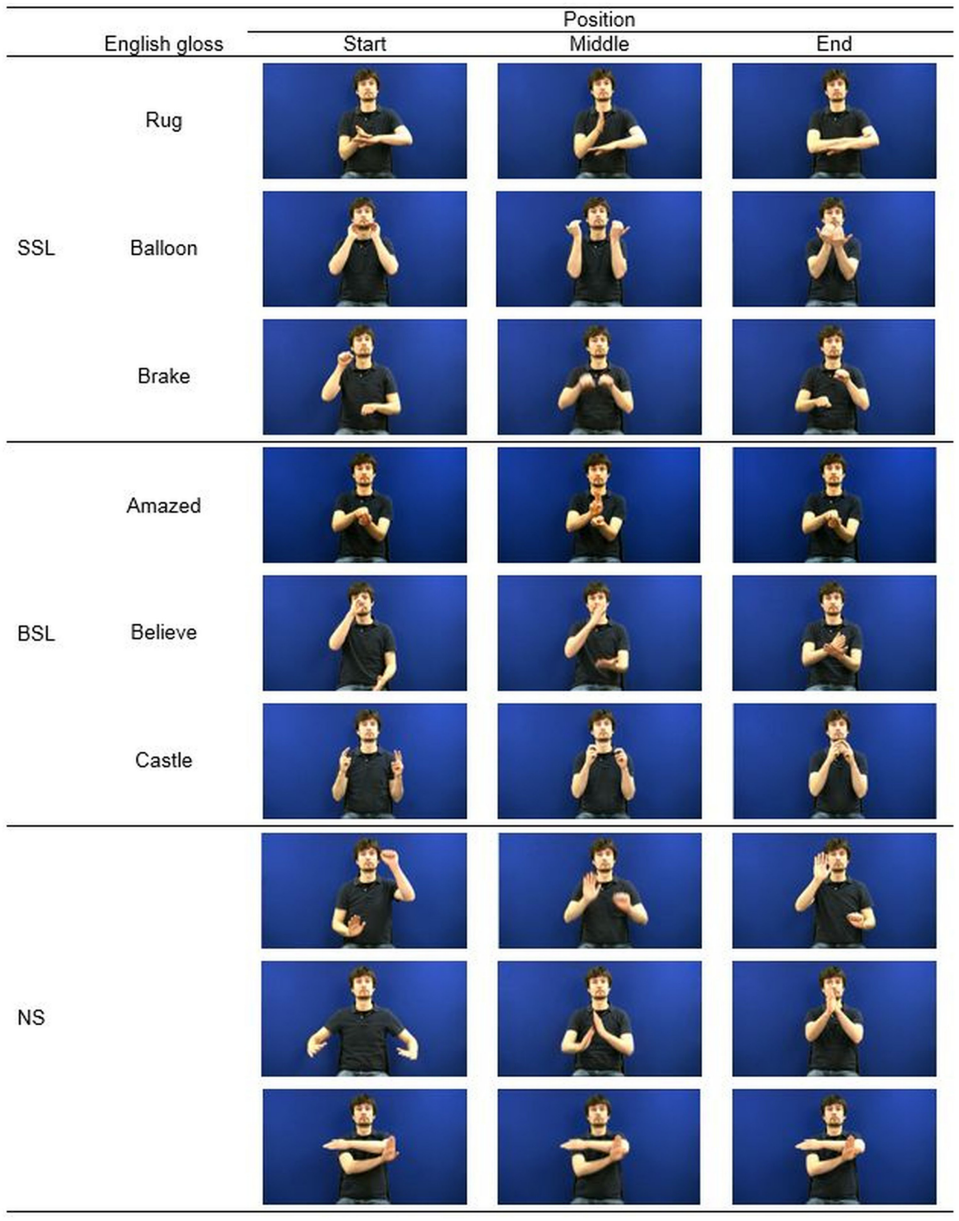
**Stimulus material for the imitation task showing still of start, middle, and end position.** SSL, Swedish Sign Language; BSL, British Sign Language; NS, Non-signs.

Stimuli were selected from an available set of videorecorded manual gestures including signs lexicalized in SSL but not BSL (chosen to be familiar to the DHH participants but not the hearing participants), signs lexicalized in BSL but not SSL (chosen to be unfamiliar to the DHH participants but phonologically plausible) and non-signs, that is manual gestures that violate the phonological rules of both sign languages or contain combinations of phonological parameters that do not occur in either language (c.f., Orfanidou et al., 2009; Cardin et al., 2016; Rudner et al., 2016). A total of nine videos of bimanual gestures were selected, three of each type (see **Figure 1**). To keep facial expressions neutral across all types of manual gestures, non-manual features of the SSL and BSL signs were not performed. Videos were of high definition quality (1080 × 720 pixels) and were presented at the center of the screen of an laptop (15.4 inches) with presentation software DMDX (version 4.1.2.0; Forster and Forster, 2003).

The order of presentation was randomized seperately on the two occasions. As an introduction to the task, the participant was given the following instruction: “Now, you are going to see some videos on the computer. In each video, there is a man who will do something. I want you to watch carefully what he does.” This instruction was given to make sure that the participant was focused on the screen before starting the test. Making sure that the participant is attentive to the target is an important part of imitation paradigms (Dickerson et al., 2013; Wang et al., 2015). When the first video had been played, the screen went blank and the child was told: “Now, it is your turn”. This comment is commonly used as a neutral prompt to elicit a response in imitation paradigms (Dickerson et al., 2013; Wang et al., 2015). If the child did not initiate an imitative act (i.e., move their hands and arms in an attempt to imitate the target) within 30 s from the point at which the video ended, the instruction (i.e., “Now, it is your turn”) was repeated once. When the child had responded the test leader clicked a button to move on to the next video. If the child did not respond within 30 s of the second instruction the test leader moved on to the next video. The same procedure was repeated for each of the remaining eight videos. Across all participants, the test leader moved on to the next video without a response being given by the child six times at T1 and two times at T2. All non-responses occurred in the DHH group.

#### Scoring

Test sessions were videorecorded and individual responses to target videos were coded at a later time. The order in which videos were coded was randomized for each rater. The coding procedure in the present study was inspired by earlier imitation paradigms (Meltzoff and Moore, 1977; Nordqvist et al., 2015), in which reliable coding typically can be achieved after a restricted amount of training. A visual analog scale (VAS, Rudner et al., 2012) was used instead of a categorical coding system (e.g., correct/incorrect) to maximize variance. The VAS was a horizontal line on a sheet of paper with fixed end points, “No correspondence” and “Perfect correspondence” but no intermediate grading. The precision of each individual response was rated by putting a corresponding cross on the VAS. The score was the proportion of correspondence, i.e. if the cross was half way along the VAS, the score was 50%, and all non-responses were scored as 0. All responses were coded independently by two trained individuals and intraclass correlation coefficients were >0.70. The dependent measure was the average between-rater score across type of gesture.

### Predictor Variables

#### Language Skills

All participants performed a phonological decision task (Cross-Modal Phonological Awareness Test) in their first language, SSL for DHH participants and Swedish for hearing participants, two Swedish word reading tasks (lexical decision and Wordchains, Jacobson, 2001), and one Swedish reading comprehension task (Woodcock Passage Reading Comprehension Test, WPRC). In addition, DHH participants performed a SSL comprehension test.

#### Swedish Sign Language Comprehension

The SSL Receptive Skills Test (see Holmer et al., 2016), an adaptation of a BSL original (Herman et al., 1999), was administered to the DHH participants as a measure of SSL comprehension. Forty videos of SSL sentences were presented one at a time to the participant who had to judge which picture out of three or four alternatives best represented the meaning of each sentence. The test was administered by trained native SSL users. One point was awarded for each correct response and the dependent measure was the number of correct responses. For two of the participants, scores pertained to testing less than 12 months before T1.

#### Cross-modal Phonological Awareness Test

The Cross-modal Phonological Awareness Test (C-PhAT; Holmer et al., 2016) was used to assess phonological awareness. The C-PhAT can be used to assess phonological awareness of both SSL and Swedish using the same materials (c.f., Andin et al., 2014). In the present study, DHH participants performed the SSL version (C-PhAT-SSL) and hearing participants performed the Swedish version (C-PhAT-Swed). In both versions, pairs of printed characters (i.e., digits and letters) were presented on a laptop (15.4 inches screen) in presentation software DMDX (version 4.1.2.0; Forster and Forster, 2003). The participant determined if the phonological labels of the printed characters were phonologically similar or not. In the SSL version this required determining whether or not they shared a handshape in the Swedish Manual Alphabet or Manual Numeral System (C-PhAT-SSL) and in the Swedish version this involved determining whether or not they rhymed in Swedish (C-PhAT-Swed), see **Table 1**. Button-press responses were given. The number of hits was adjusted for false alarms in accordance with signal detection theory (Swets et al., 1961); thus, *d’* was the dependent measure on both versions of the task.

**Table 1:** Examples of pairs in the Cross-Modal Phonological Awareness Test that have similar phonological labels in Swedish (Category 1); in the Swedish manual alphabet or manual numeral systems (Category 2); and in neither (Category 3).

	Category					
	
	1		2		3	
Print	5	M	S	C	T	U
Swedish	/fεm/	/εm/	/εs/	/ce:/	/te:/	/ʉ:/
SMS	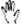	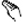	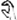	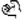	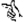	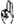


*SMS, Swedish Manual Alphabet and Manual Numeral System.*

#### Word Reading

Two measures of word reading were administered to both groups. The first task was a lexical decision task, in which participants were presented with three-letter items in lowercase on a laptop (15.4 inches screen) with presentation software DMDX (version 4.1.2.0; Forster and Forster, 2003). Items were real words, pseudo-words (i.e., items that are pronouncable and look like real Swedish words but lack meaning) and non-words (i.e., items that cannot be real words in Swedish) presented one at a time on the screen in a set order and the participant decided, for each item, if it was a real word in Swedish or not. There were 20 real words, 10 pseudo-words, and 10 non-words. Responses were made by pressing buttons corresponding to yes or no. The time limit for a response was 20 s, and between items the screen went blank for 1 s. The dependent measure was *d’* (Swets et al., 1961).

The second task that was used to assess word reading was Wordchains (Jacobson, 2001), an established test in the Nordic countries (e.g., Asbjørnsen et al., 2010). In this task, the participant was presented with uninterrupted strings of characters that could be separated by pen strokes into three different Swedish words, e.g., hej|mat|snö (in English, hi| food|snow). In total, there was 60 different wordchains evenly distributed on 20 rows on a sheet, and the participant had 2 min to solve as many chains as possible. The participant practiced the task with three separate chains and was instructed how to correct an erronous response before testing commenced. The dependent measure was the number of chains correctly completed within the two minute time limit. The two tests of word reading were combined into a word reading score, by converting raw data to normal scores and then averaging the normal scores into one single variable.

#### Woodcock Passage Reading Comprehension

The Swedish version of the WPRC test (Furnes and Samuelsson, 2009) was used as a measure of Swedish language comprehension. In this test, passages of text of different length in which one word is omitted were presented to the participant. Hearing participants had to say or write a word that completed the passage; DHH participants could answer by providing an appropriate sign or, saying or writing a word. At the beginning of the test, passages consist of single three-word sentences and at the end of the test, passages include several sentences with both main and subordinate clauses. Testing was stopped after a sequence of six consecutive errors. In total there were 68 passages, and the dependent measure was the number of correct answers.

#### Motor Skill

To assess motor control, a bead threading task was used (White et al., 2006). Participants threaded nine colored wooden beads of different shapes onto an 8 mm thick string with a knot in the end. The task was administered twice and the participants were asked to thread the beads onto the string as fast as possible. The fastest completion time in s across the two trials was the dependent measure.

#### Working Memory

The Clown test (Sundqvist and Rönnberg, 2010; Birberg Thornberg, 2011), based on the Mr. Peanut task (Kemps et al., 2000), was used as a measure of visual working memory. A clown figure on a magnetic board with varying numbers of magnets placed at different locations was shown to the participant. The figure was then turned away from the participant, the magnets were removed, and the participant had to say the color of the magnets. After that, the figure was once again turned towards the participant who was given the magnets and instructed to reproduce the pattern presented earlier. The number of magnets increased from one at the first level, up to a maximum of ten. There were three trials on each level and on each trial the magnets were all of the same color (red, blue, or yellow) and placed in a pre-defined order. Two incorrect answers on one level led to discontinuation of the task. One point was awarded for each correct trial, and the dependent measure was total score.

### Data Analysis

First, descriptive statistics were calculated and between group differences were investigated. In a second step, a repeated measures split-plot ANOVA was conducted with two within group factors: occasion with two levels (T1, T2), and type of manual gesture with three levels (SSL, BSL, non-signs), and one between group factor with two levels (DHH, Hearing). *Post hoc* analyses and exploration of simple main effects were then performed. In the final step, correlational analysis of relations between predictor variables (SSL comprehension, NVIQ, Working memory, Bead threading, C-PhAT, Word reading, and WPRC) at T1 and imitative ability (average score across all responses) at both occasions was conducted.

Some violations of normality were detected on the predictor variables in the hearing group. Thus, parametric and non-parametric methods for between group comparisons and correlations were compared in analyses involving these measures. No differences were detected between approaches and therefore we only report results from parametric methods (i.e., *t*-tests and Pearson *r*). A two-tailed significance level of 0.05 was applied, and to obtain maximum power, despite low *n*, no correction was made for multiple comparisons. Descriptive statistics, correlations and the split-plot ANOVA, with *post hoc* tests, were conducted using IBM SPSS Statistics (Version 22.0), and simple main effects were calculated manually in Microsoft Excel (2013) following the recommendations of Kirk (1994).

### Missing Data

For one DHH participant all responses on the imitation task were missing at both occasions. In addition, a full set of responses on the same task was missing from another DHH participant at T1 and one further DHH participant at T2. One full set of imitation responses was also missing from one hearing participant at T2. All these responses were missing due to technical errors. In addition, one further hearing participant failed to perform the imitation task at T2. A number of responses were coded as missing because they were performed out of picture. This applied to three responses from one DHH participant at T1, and one response each from another DHH participant and two hearing participants at T2. Finally, one DHH participant did not do the test of SSL comprehension.

When calculating average imitation scores on the three types of manual gestures (SSL, BSL, and non-signs) and the average imitation score across all items in the task, all available data for each individual was used. In statistical analyses, the missing completely at random (MCAR) mechanism was assumed, i.e., absence of data was assumed to be entirely haphazard (Enders, 2010). Listwise (in ANOVA) or pairwise (in correlations and regression) deletion were used to handle missing data, since these procedures provide unbiased estimates under the MCAR mechanism (Enders, 2010).

## Results

### Descriptive Statistics

There were no differences between groups on gender distribution, χ^2^(1) = 0.01, *p* = 0.92, NVIQ, Working memory, Bead threading, or Word reading (see **Table 2**). DHH participants were older than hearing participants, *t*(12.2) = 4.0, *p* = 0.002, but performed worse than them on WPRC (see **Table 2**). Girls outperformed boys on Bead threading at both occasions in both groups (*p*s < 0.05). No other gender differences were revealed (*p*s > 0.05). Age and NVIQ were unrelated to performance on the imitation task in both groups (*p*s > 0.05).

**Table 2:** Descriptive statistics and between group *t*-tests for predictor variables.

	DHH (*N* = 13)			Hearing (*N* = 36)			
			
Measures	*M*	*SD*	95% CI	*M*	*SD*	95% CI	*t*-test
SSLC^a,b^	33.0	5.15	[29.7, 36.3]	–	–	–	–
NVIQ^b^	25.2	5.88	[21.7, 28.8]	25.4	4.35	[23.9, 26.9]	ns
WM^b^	2.08	0.67	[1.67, 2.48]	1.83	0.82	[1.55, 2.11]	ns
BT	34.0	8.69	[28.8, 39.3]	33.7	7.96	[31.0, 36.4]	ns
C-PhAT^c^	1.03	1.22	[0.29, 1.76]	2.17	1.22	[1.76, 2.58]	–
WC^b^	7.23	4.76	[4.35, 10.1]	8.28	4.35	[6.81, 9.75]	ns
LD^b,c^	0.39	0.57	[0.05, 0.73]	0.47	1.03	[0.12, 0.81]	ns
WPRC	3.77	1.24	[3.02, 4.52]	13.5	8.77	[10.5, 16.5]	*P* < 0.001


*DHH, Deaf and hard-of-hearing; SSLC, Swedish Sign Language comprehension (raw score); NVIQ, Non-verbal intelligence (raw score); WM, Working memory (raw score); BT, Bead threading; C-PhAT, Cross-Modal Phonological Awareness Test, SSL version (C-PhAT-SSL) for DHH participants and Swedish version (C-PhAT-Swed) for hearing participants (*d’* scores); WC, Wordchains (raw score); LD, Lexical decision (*d’* scores); WPRC, Woodcock Passage Reading Comprehension (raw scores).*

*^a^*n* = 12.*

*^b^Data also reported in Holmer et al. (2016).*

*^c^*d’*, a value > 0 indicates better than chance performance.*

### Imitation Task

**FIGURE 2:**
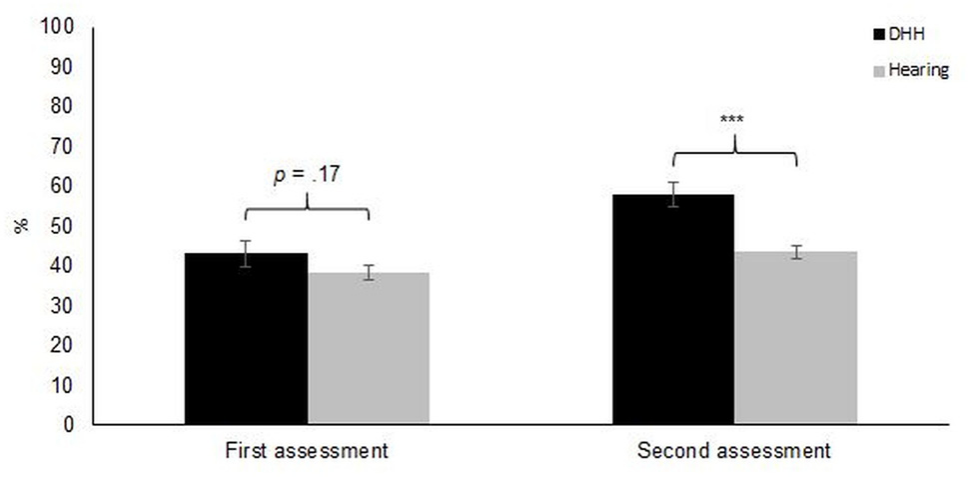
**Overall performance on the imitation task (average score across all available items; 100 on the Y-axis represents ratings of perfect correspondence between target and response) for deaf and hard-of-hearing (DHH) and hearing participants at T1 and T2.** Error bars represents ±1 SE. ^∗∗∗^*p* < 0.001.

Performance on the imitation task is presented in **Table 3**. In the split-plot ANOVA, the assumption of sphericitiy was satisfied and error variances were homogeneous on imitation of all types of gestures across groups. The main effects were statistically significant: occasion, *F*(1,42) = 45.5, $\eta_{p}^{2}$ = 0.52, *p* < 0.001; type of manual gesture, *F*(2,84) = 4.74, $\eta_{p}^{2}$ = 0.10, *p* = 0.011; and group, *F*(1,42) = 8.27, $\eta_{p}^{2}$ = 0.16, *p* = 0.006; as well as the group by occasion interaction, *F*(1,42) = 10.7, $\eta_{p}^{2}$ = 0.20, *p* = 0.002 (see **Figure 2**). The group by type of manual gesture interaction was not significant, *F*(2,84) = 0.96, $\eta_{p}^{2}$ = 0.02, *p* = 0.39, disfavouring our initial prediction that DHH signing would perform better on the SSL signs than both on the BSL and non-signs. All other interactions were also non-significant (*p*s > 0.05). Removing the non-responses of DHH participants from the imitation scores did not change the results.

**Table 3:** Performance on the imitation task at T1 and T2 for deaf and hard-of-hearing and hearing participants.

	T1						T2					
		
	DHH (*N* = 13)^a^			Hearing (*N* = 36)			DHH (*N* = 13)^a^			Hearing (*N* = 36)^a^		
				
	*M*	*SD*	95% CI	*M*	*SD*	95% CI	*M*	*SD*	95% CI	*M*	*SD*	95% CI
Total	43.3	13.9	[33.9, 52.6]	38.1	9.35	[34.9, 41.3]	59.0	11.7	[51.1, 66.9]	43.6	8.81	[40.6, 46.7]
SSL	47.0	15.6	[36.6, 57.5]	37.7	11.6	[33.8, 41.7]	57.8	12.1	[49.7, 66.0]	43.2	12.4	[38.9, 47.5]
BSL	44.9	16.7	[33.7, 56.1]	41.0	12.9	[36.7, 45.4]	62.3	12.7	[53.7, 70.8]	45.1	12.1	[40.9, 49.3]
Non-signs	37.0	14.8	[27.1, 47.0]	35.6	12.5	[31.4, 39.9]	56.6	17.3	[45.0, 68.2]	42.7	11.7	[38.6, 46.8]


*DHH, Deaf and hard-of-hearing; Total, Average score across all manual gestures; SSL, Average score on manual gestures that are lexicalized in SSL; BSL, Average score on manual gestures that are lexicalized in British Sign Language; Non-signs, Average score on manual gestures that violate phonotactical rules of SSL and BSL.*

*^a^Two missing cases.*

**FIGURE 3:**
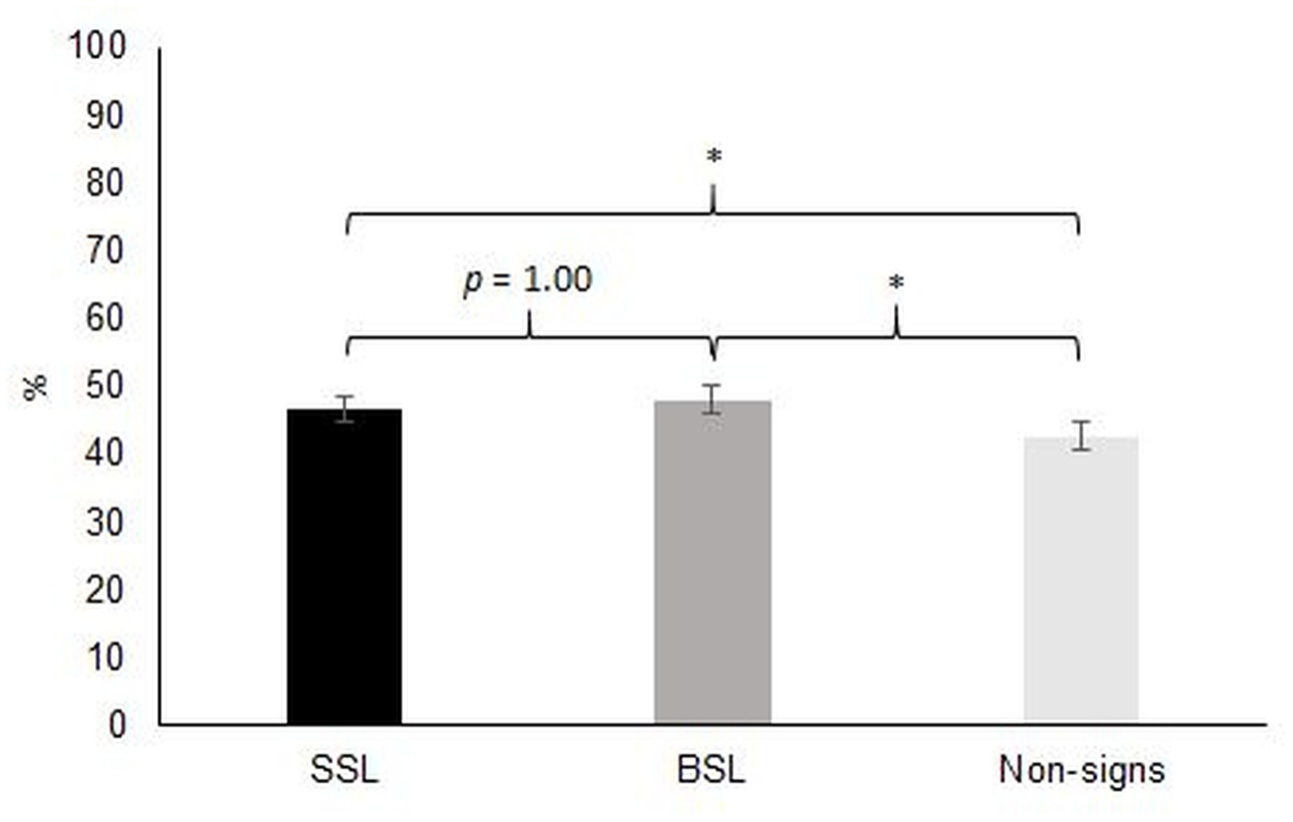
**Overall performance across all participants on the three levels of type of manual gesture (SSL, BSL, and non-signs) in the imitation task (average score across all available items; 100 on the Y-axis represents ratings of perfect correspondence between target and response).** Error bars represents ±1 SE. SSL, Swedish Sign Language; BSL, British Sign Language.

*Post hoc* analyses of the main effects revealed that performance was better at the second occasion (T2) than at the first occasion (T1), mean difference = 10.0, and that DHH participants outperformed hearing participants, mean difference = 9.50. The mean differences across groups between imitation of SSL and of non-signs (4.24), as well as between imitation of BSL and of non-signs (5.55) were statistically significant, showing that imitation of non-signs was poorer than imitation of both SSL and BSL signs. However, there was no difference in performance between SSL and BSL (see **Figure 3**). Simple main effects of the group by occasion interaction revealed that the performance of both DHH, *F*(1,9) = 10.9, *r* = 0.72, *p* = 0.009, and hearing participants, *F*(1,33) = 4.46, *r* = 0.34, *p* = 0.042, improved over time. Further, the DHH group outperformed the hearing group at T2, *F*(1,45) = 19.0, *r* = 0.55, *p* < 0.001, but not T1, *F*(1,45) = 1.96, *r* = 0.20, *p* = 0.17. Thus, in contrast to what was predicted, the DHH group did not have an initial advantage on the task, nor did hearing participants have a steeper development between the two occasions than did DHH children. Rather, DHH children showed a stronger development than hearing children, as evident from the significant group by occasion interaction.

### Predicting Performance on the Imitation Task

The correlations between predictor variables (NVIQ, SSL comprehension, Working memory, Bead threading, Cross-modal Phonological Awareness Test, Word reading, and WPRC) at T1 and performance on the imitation task at both occasions were explored to investigate our predictions (see **Table 4**). For DHH participants, imitative precision at T1 predicted imitative precision at T2, *r*(10) = 0.65, *p* = 0.040. Partial support for our initial prediction that sign language skills should be related to imitative ability was found in the pattern of correlations. Word reading at T1 was significantly associated with imitative ability at both T1, *r*(11) = 0.70, *p* = 0.016, and T2, *r*(11) = 0.80, *p* = 0.003. Further, performance on the imitation task at T2 was predicted by SSL comprehension, *r*(11) = 0.70, *p* = 0.017, and phonological awareness, *r*(11) = 0.64, *p* = 0.035, at T1. Excluding non-responses from imitation scores did not affect the correlational pattern.

**Table 4:** Correlations between predictor variables at T1 and performance on the imitation task at both T1 and T2.

		Imitation			
		
		DHH (*N* = 13)		Hearing (*N* = 36)	
			
		T1^a^	T2^a^	T1	T2^a^
T1	WR	0.70^∗^	0.80^∗∗^	0.03	0.20
	WPRC	0.50	0.21	0.17	0.43^∗^
	SSLC	0.16	0.70^∗^	–	–
	C-PhAT	0.53^†^	0.64^∗^	0.20	0.25
	WM	0.20	0.40	0.23	0.14
	BT	-0.18	-0.09	-0.29	-0.21
	NVIQ	-0.12	0.41	0.18	0.13


*DHH, Deaf and hard-of-hearing; WR, Word reading; WPRC, Woodcock Passage Reading Comprehension; SSLC, Swedish Sign Language comprehension; C-PhAT, Cross-Modal Phonological Awareness Test, SSL version (C-PhAT-SSL) for DHH participants and Swedish version (C-PhAT-Swed) for hearing participants; WM, Working memory; BT, Bead threading; NVIQ, Non-verbal intelligence.*

*^a^Two missing cases.*

*^∗^*p* < 0.05, two-tailed. ^∗∗^*p* < 0.01, two-tailed. ^†^*p* < 0.05, one-tailed.*

As for DHH participants, imitiative precision at T1 was related to imitative precision at T2 for hearing participants, *r*(34) = 0.66, *p* < 0.001, indicating stability in performance on the imitation task over time for both samples. Further, for the hearing participants, scores on WPRC at T1 predicted performance on the imitation task at T2, *r*(34) = 0.43, *p* = 0.012. Thus, the overall pattern indicates a connection between language and imitation of manual gestures. However, connections are more broadly distributed for DHH signing than for hearing non-signing children.

To test the predictive power of language comprehension on imitative ability at T2 for hearing participants, a hierarchical regression model was conducted. In the first step, imitative ability was regressed on itself. In a second step, Bead threading and Working memory was included, to control for variance accounted for by motor skills and working memory. In the third and final step, WPRC was added as a predictor (see **Table 5**). The addition of WPRC led to a Δ*R*^2^ of 0.09 which was significant, *F*(1,31) = 5.83, *p* = 0.022, and the final model explained 49.9% of the variance in imitative ability at T2, *F*(4,31) = 7.72, *p* <0.001. Errors were normally distributed and inspection of the scatterplot between residuals and predicted values indicated homoscedasticity.

**Table 5:** Hierarchical regression model for predicting performance of hearing participants on the imitation task at T2.

	*R^2^*	Δ*R*^2^	β	*t*	*p*
Step 1: Regressing on initial level of imitative ability					
Imitation at T1 (average score across all responses)			0.59	4.38	<0.001
	40.3%	40.3%			
Step 2: Cognitive and motor control variables entered					
Working memory (raw score)			-0.02	0.18	0.860
Bead threading (in *s*)			0.04	0.25	0.805
	40.5%	0.20%			
Step 3: Language comprehension variable entered					
Woodcock Passage Reading Comprehension (raw score)			0.32	2.42	0.022
	49.9%	9.42%			


## Discussion

In the present study, we elicited imitation of manual gestures from Swedish DHH signing children and hearing non-signing children at similar levels of cognitive and language development, with the aim of studying how pre-existing linguistic knowledge influences precision of imitation. We predicted that the DHH signing children would be better at imitating manual gestures lexicalized in their own sign language (SSL) than unfamiliar BSL signs, and that both groups would be better at imitating lexical signs (SSL and BSL) than non-signs. We also predicted that the hearing non-signing children would perform worse than DHH signing children with all types of gestures the first time we elicited imitation, but that the performance gap between groups would be reduced when imitation was elicited a second time. Finally, we predicted that imitation performance on both occasions would be associated with linguistic skills, especially in the manual modality.

### No Effect of Familiarity

Contrary to our prediction, we did not find any evidence that pre-existing knowledge of SSL improved precision of imitation of signs lexicalized in SSL compared to signs lexicalized in another sign language (BSL) for the DHH signing participants. We derived our prediction from the ELU model, which states that language processing is rapid and automatic if input matches pre-existing phonological and semantic representations (Rönnberg et al., 2013). We reasoned that because, for the DHH signing participants, the repertoire of phonological components is similar for SSL and BSL (Rudner et al., 2016), the unfamiliar BSL signs would match existing phonological representations. However, because the cohort of lexical candidates activated by BSL signs would not be constrained by meaning (Marslen-Wilson, 1987), our assumption was that a better match would be obtained with SSL signs than with BSL signs, leading to better imitation for the DHH signing participants.

It is possible that the three specific SSL items chosen in the present study from SSL did not match the existing representations of the DHH signing participants because they had not yet been acquired. However, we deem this unlikely as the items were commonly occurring. Another possibility is that the number of participants and the number of trials were too small to detect this effect. However, this is also unlikely because the experiment was repeated on a second occasion. Thus, the present results strongly suggest that in DHH signing children who are at an early stage of their reading development, pre-existing semantic representation does not enhance imitation more than pre-existing phonological representation. There are examples relating to deaf signing adults of pre-existing semantic representation not influencing either behavior (Rudner et al., 2016) or neural processing (Petitto et al., 2000; Cardin et al., 2016), and it has been argued that this may by due to the fact the phonology of sign language often carries semantic information (Thompson et al., 2012). One interpretation of the absence of an effect of sign familiarity in the present study is that for sign language users, semantic representation does not constrain the cohort of lexical candidates activated by phonologically plausible exemplars. Thus, sign-related semantic representation may not play the same role as speech-related semantic representation in the mechanism described by the ELU model (Rönnberg et al., 2013).

It is important to note that the target items used in the present study did not include non-manual gestures. Non-manual aspects of lexical signs may be important for achieving a match between an incoming signal of degraded quality and existing representations in the mature mental lexicon (Quer and Steinbach, 2015) and thus contribute to ease of language understanding. Such an effect is likely to be even more important in the developing language system. Thus, future work should investigate the role of non-manual components in the ability of DHH signing children to imitate signs in their own and unfamiliar sign languages.

### Effect of Lexicality

Recent studies indicate that even for non-signers, non-signs are more difficult to process than lexical signs (Cardin et al., 2016; Rudner et al., 2016). This suggests that it is more demanding to process manual gestures that break the phonological rules of signed languages, even for individuals with no previous knowledge of sign language. The implication of this is that the phonological characteristics of a language may arise as a consequence of more efficient neural processing for its perception and production. Thus, we predicted that in the present study, both groups would be better at imitating lexicalized signs (both SSL and BSL) than non-signs. This was exactly what we found.

Other work indicates that it is easier to imitate meaningful acts (e.g., pantomimes of object use) than novel, meaningless acts (Tessari and Rumiati, 2004), and it has also been suggested that imitation builds on understanding intent and goal-directedness of an action (Bekkering et al., 2000; Want and Gattis, 2005). Thus, more precise imitation of lexical signs than non-signs in the present study may be driven by differences in the perceived meaningfulness, intent and goal-directedness of the items as well as in inherent motor patterns. Future work should use sign-based stimuli generated by computerized avatars to separate the effects of phonologically legal motor patterning on the one hand and meaningfulness, intent and goal-directedness on the other.

Surprisingly, the DHH signing children were no more precise in their imitation of lexical signs than the hearing non-signing children. The inability to find any difference between groups, might in part be due to statistical issues relating to diverging variances across groups or the form of distributions on variables. However, statistical tests indicated equal variances across groups as well as normally distributed imitation scores, indicating that these factors did not influence results, although it should be noted that the power to detect such violations was restricted. Thus, we found no evidence to support the notion that pre-existing phonological representation facilitates imitation of unfamiliar but phonologically acceptable manual gestures, but we cannot rule out that this may have been due in part to methodological issues.

### Effect of Prior Imitation

Both groups were more precise in their imitation of manual gestures second time round. We had predicted that the increment would be greater for hearing children than for the DHH signing children. This prediction was based on the notion that preexisting representation would facilitate language processing, in line with the ELU model (Rönnberg et al., 2013). Specifically, we predicted that the DHH group would have an advantage over the hearing group at the first occasion (T1). However, we predicted that this advantage would diminish at the second occasion (T2) because the hearing children would be able to make use of the representations they had encoded into episodic long-term memory at T1. However, the opposite was true. While there was no difference between groups in precision of imitation at T1, the DHH group produced more precise imitations at T2 than the hearing children. This fits in with the lack of evidence that pre-existing linguistic representation facilitated imitation.

The pattern of results suggests that T1 provided an opportunity for both groups to establish representations that they could exploit at T2. The fact that the improvement in imitation over time did not interaction with stimulus type strengthens the notion that pre-existing linguistic representation does not support imitation and suggests that the improvement in imitation performance at T2 was driven by the ability to form item-specific representations, irrespective of lexiciality. However, the fact that the DHH group showed a greater improvement in imitation ability over time suggests that they were more successful than the hearing group in exploiting those item-specific representations.

### Correlations with Language Skills

Language skills assessed at T1 predicted precision of imitation at T2 for both groups. In particular, for the DHH group, SSL phonological awareness measured using the C-PhAT (Holmer et al., 2016), SSL proficiency, measured using a SSL comprehension test, and Swedish word reading all strongly predicted precision of imitation at T2. Imitation at T1, however, was only significantly correlated with word reading, although the correlation with SSL phonological awareness was also marginally significant. This pattern of correlations, suggests that SSL skills, including phonological awareness and comprehension, are mobilized during imitation, but only when adequate representations have already been established. Further, the correlation with word reading may also suggest mobilization of sign language skills, as written words seem to be recoded to their corresponding signs in DHH signers (Leinenger, 2014). The relation between sign language skills and imitation of manual gestures, should be investigated in larger samples in future studies.

For the hearing group, reading comprehension at T1, a proxy for speech-based representation, correlated significantly with precision of imitation at T2, whereas none of the language variables correlated with precision of imitation at T1. Indeed, regression analysis showed that reading comprehension at T1 explained unique variance in imitation precision at T2, above and beyond variance explained by imitation precision at T1, motor skill and working memory. This suggests hearing non-signing children mobilize language comprehensions skills during imitation of manual gestures, rather than motor skills or working memory, but only when adequate representations have already been established. Taken together, the pattern of correlations across groups provides support for our prediction of a positive relationship between imitation and linguistic skills, especially in the manual modality.

### Overall Interpretation

The specific predictions relating to the influence of pre-existing semantic and phonological representation on precision of imitation were based on the limited number of studies performed to date. In any small field, the results of any new study may be at least partly unexpected and that was the case here. The pattern of results revealed by the present study suggests that for children whose language skills are still developing, the establishment of item-specific representations of manual gestures is supported by both domain general and modality specific skills. Specifically, DHH signing children seem to be able to make use of modality specific language skills, although not pre-existing linguistic representations, to establish new representations of manual gestures, while establishment of manual representations in hearing non-signing children seems to be supported by the domain general aspect of language processing.

**FIGURE 4:**
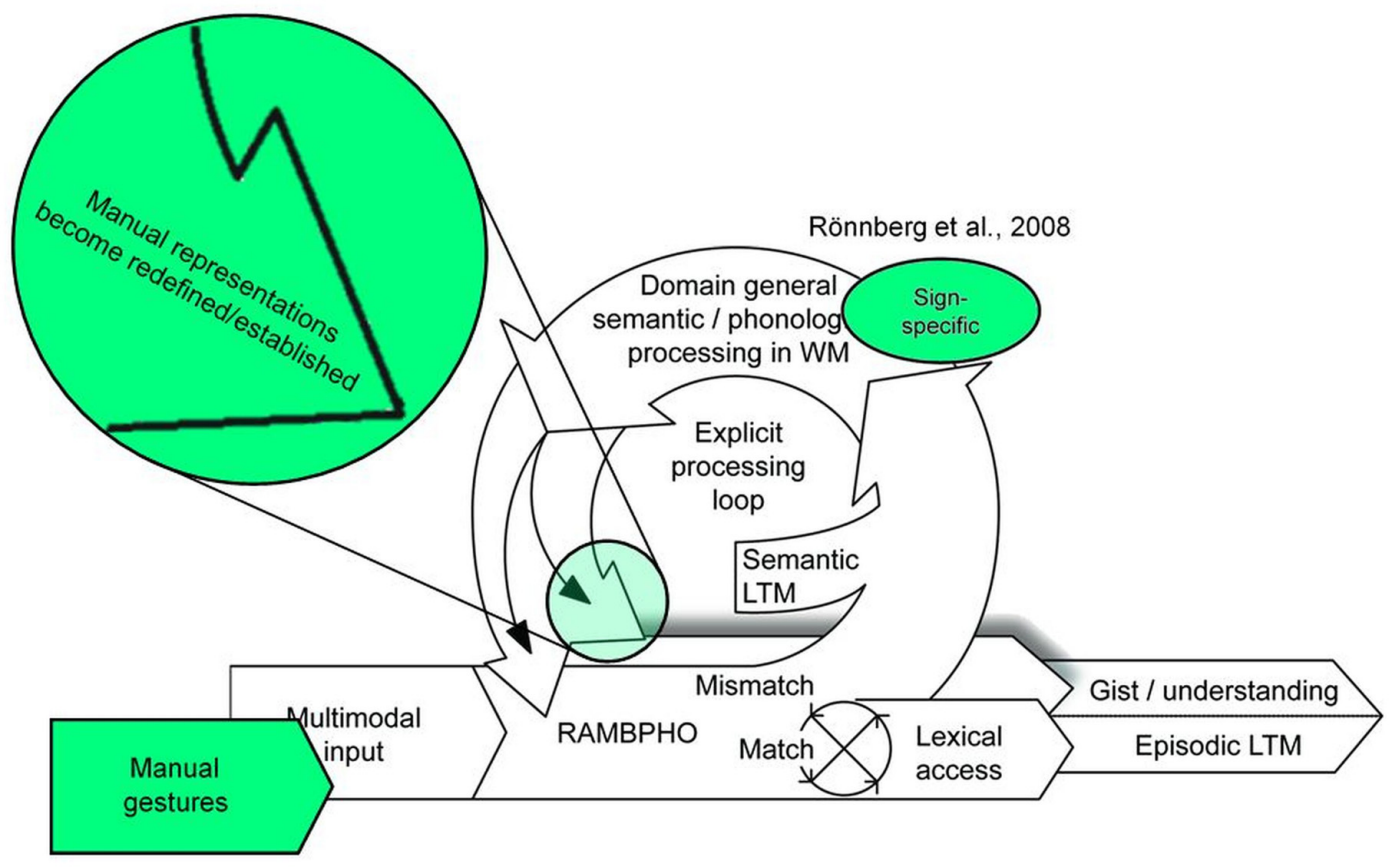
**The Developmental Ease of Language Understanding (D-ELU) model.** Modifications to the ELU model (Rönnberg et al., 2013) are marked with green. The modality specific content in the explicit processing loop is reintroduced from Rönnberg et al. (2008). Adapted from “The ELU model: Theoretical, empirical, and clinical advances” by Rönnberg et al. (2013). Copyright 2013 by Rönnberg, Lunner, Zekveld, Sörqvist, Danielsson, Lyxell, Dahlström, Signoret, Stenfelt, Pichora-Fuller and Rudner under the CC BY 3.0 license (http://creativecommons.org/licenses/by/3.0/).

These modality-specific findings suggest that the ELU model (Rönnberg et al., 2013) cannot be applied directly to sign language, at least with reference to the developing language system. Hence, we suggest a modified version of the ELU model, i.e., a D-ELU model (see **Figure 4**). Like ELU, D-ELU emphasizes the importance of a good match between language input and pre-existing representations for language formation. However, whereas ELU predicts domain specific explicit processing when there is a mismatch between input and existing representations, D-ELU predicts that when there is a mismatch between input signal and stored linguistic representations in the developing language system, the explicit processing loop engages both domain general representations (e.g., semantic long-term memory) and domain specific representations (e.g., sign-specific phonology) in the analysis of the incoming language signal. This process leads to establishment of new representations or a redefinition of stored representations, a notion in line with perceptual magnet theory (Kuhl, 1991) which predicts a warping of the perceptual space around phonological representations as learning progresses. In comparison to the mature language system which is more tolerant of phonological diversity, this process is qualitatively different. Thus, an adaptation of the ELU model for the developing language system is warranted. Interestingly, changes in phonological representation are also characteristic of individuals with post-lingual hearing loss (Classon et al., 2013). Thus, One possibility is that D-ELU could also help us understand ELU towards the end of the lifespan. In order to account for the lack of interaction between phonology and semantics in sign language processing, reported both here and in earlier studies (Cardin et al., 2016; Rudner et al., 2016), a sign specific component should be reintroduced into the model (c.f., Rönnberg et al., 2008). Future work should test the generalizability of the proposed D-ELU model by investigating the role of language skills across modalities in establishment of linguistic representations.

## Conclusion

The act of imitation allows both DHH signing and hearing non-signing children to establish specific representations which together with language skills facilitate future imitation. This set of findings prompts an adaptation of the ELU model, D-ELU.

## Author Contributions

EH, MH, and MR designed the study. EH co-ordinated data collection and coding, and performed the statistical analyses. EH prepared the first draft of the article and all authors contributed to the final version.

## Conflict of Interest Statement

The authors declare that the research was conducted in the absence of any commercial or financial relationships that could be construed as a potential conflict of interest.
